# From molecules to medicine: a systematic review of Gastrodia elata’s bioactive metabolites and therapeutic potential

**DOI:** 10.3389/fphar.2025.1641443

**Published:** 2025-11-07

**Authors:** Xiantai Zhou, Minna Han, Shiyu Yan, Mengyuan Wang, Shengzhou Zu, Shenghui Zhong

**Affiliations:** 1 Southern Medical Branch of PLA General Hospital, Beijing, China; 2 Department of Pharmacy, Guilin Medical University, Guilin, Guangxi, China; 3 School of Basic Medical Sciences, Yichun University, Yichun, Jiangxi, China

**Keywords:** Gastrodia elata, biological activity, chemical composition, pharmacological effects, treatment ofdiseases, pharmacokinetics

## Abstract

The dried tuber of Gastrodia elata (GE), a perennial orchid with a 2,200-year medicinal history documented in the Shennong Bencaojing (200 BCE), remains a cornerstone of traditional Chinese medicine (TCM) and contemporary integrative therapies across Asia. Initially prescribed for neurological disorders (e.g., epilepsy, stroke prophylaxis) and hypertension, modern research has expanded its therapeutic portfolio to include anti-aging, antitumor, and osteoprotective applications. This systematic review synthesizes 1) traditional ethnopharmacological uses, 2) phytochemical profiling of 100+ identified bioactive metabolites (e.g., gastrodin, parishins), and 3) mechanistic insights into their pharmacokinetic behaviors and pharmacodynamic actions. Notably, botanical drug interactions in TCM formulations enhance gastrodin’s blood-brain barrier penetration, elucidating clinical efficacy. While *in vitro*/*vivo* studies validate GE’s antioxidant and neuroprotective effects, translational challenges persist: 1) Limited clinical trials on novel indications (e.g., osteoporosis); 2) Unclear structure-activity relationships of minor metabolites; 3) Standardization needs for industrial applications. This work provides an evidence base to guide future research on GE’s diversified therapeutic development.

## 1 Introduction

As early as several thousand years ago, the Chinese people recognized that certain natural plants had better therapeutic effects in the treatment of diseases, and had many advantages such as abundant resources and ease of cultivation (Martins and S, 2018; Zhao et al., 2012). In ancient China, based on the experience of using medicinal botanical drugs, natural plants with medicinal properties were compiled into books such as “Shennong’s Classic of Materia Medica,” “Newly Revised Materia Medica,” and “Compendium of Materia Medica.” Various plants were classified according to experience and used for targeted treatment of various diseases (Hu et al., 2019; Tang et al., 2017; Tian et al., 2022). Taking Tianma (*Gastrodia elata*) as an example, the earliest recorded pharmacological work on Tianma is in “Shennong’s Classic of Materia Medica.” Over 2000 years ago, Tianma was already used for the treatment of cardiovascular, neurological, and nervous system diseases. In addition, a large amount of basic research and clinical studies have shown that Tianma can improve hemodynamics, increase blood flow, enhance vascular elasticity, and have a certain protective effect on the cardiovascular and cerebrovascular systems (Zuowei, 2017).

**FIGURE 1:**
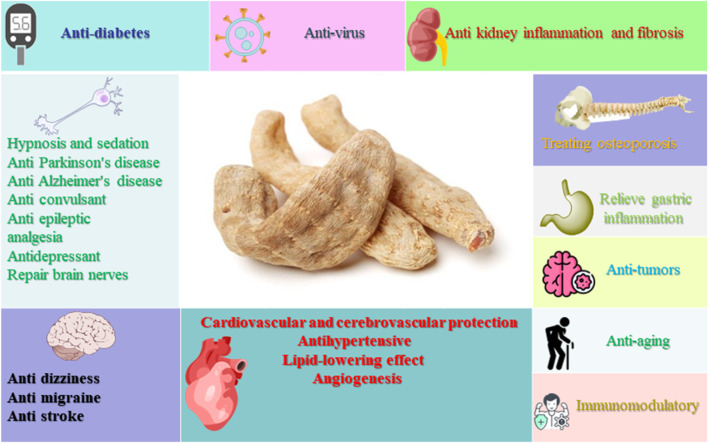
Pharmacological effects of GE.

*Gastrodia elata* is commonly called Tian ma, Red arrow, Dingfeng grass, etc., in China. *Gastrodia elata* has long been widely studied as a traditional botanical medicine in Asia, especially in China, Korean peninsula, Japan and Russia (Zhan et al., 2016). *Gastrodia elata* has a wide range of medicinal value and remains popular in Asia. In traditional Chinese Medicine, Rhizoma Gastrodiae is mainly used for hypertension, headache, stroke, limb numbness, hemiplegia, arthritis and other symptoms (Liu et al., 2018; Wang P. H. et al., 2016; Lee et al., 2012). However, with further research, researchers have found that gastrodia also has extensive pharmacological activity such as anti-tumour, anti-aging, improved memory, anti-depression, anti-insomnia and so on (Liu et al., 2018; Farooq et al., 2019; Chen and Sheen, 2011; Liang et al., 2017). The pharmacological activity of gastrodia are shown in Figure 1.

Since the beginning of this century, pharmacologically activemetabolites have been found one after another from Rhizoma Gastrodiae. Clinical studies on gastrodin have been carried out because of its definite therapeutic effect and high safety (Kong et al., 2022; Guo et al., 2015). Although gastrodia has been studied for thousands of years, its active metabolites and pharmacological effects are still waiting for being explored.

In this review, We conducted searches across multiple databases and literature sources, including PubMed, CNKI (China National Knowledge Infrastructure), Chinese Pharmacopoeia, and China Medical Information Platform. The search terms encompassed Gastrodia elata, Rhizoma Gastrodiae, Gastrodin, Neuroprotection, Anti-inflammatory, Immunomodulation, and related keywords and summarized the progress of research on the botanical, phytochemical and pharmacological aspects of *Gastrodia elata*, in the hope of providing supporting information for the in-depth development and utilisation of *Gastrodia elata*.

## 2 Botanical characteristics of Gastrodia elata

Rhizoma Gastrodiae is the dried tuber of *Gastrodia elata* Blume of the *orchid* family, which is a perennial parasitic botanical drug. Currently, gastrodin is recognized as one of the most crucial active monomers in Gastrodia elata Blume. *Gastrodia elata* cannot carry out photosynthesis because it has no chlorophyll. The seeds need to co-exist with *mosmundicola* belonging to *tricholomataceae* for providing nutrition during germination, and after growing to the protocorm, it needs to be associated with honey mushroom of the *tricholomidae* family to provide nutrition (Chen L. et al., 2019). They grow in the thick humus under the trees at an altitude of 400–3200 m, and are widely distributed in the tropical, subtropical, temperate and cold temperate mountains. Currently, there are mainly five kinds of medicinal *Gastrodia elata* (Zhan et al., 2016; Zhengyi, 1999) (Table 1). *GE Bl. form. glauca S. Chow and GE f. elata* have been widely cultivated due to their high adaptability as well as high output.

**TABLE 1:** Commonly used medicinal GE.

No	Latin name	Plant height	Flowering period	Water content of tubers
1	*GE Bl. f. elata*	1.5–2 m	4–5 months	85%
2	*GE Bl. f. viridis*	1–1.5 m	6–7 months	70%
3	*GE Bl.f.glauca S.Chow*	1.5–2 m	6–7 months	60%–70%
4	*GE Bl.f.alba S.Chow*	≈1 m	4–5 months	90%
5	*GE Bl.f.flavida S.Chow*	>1 m	4–5 months	80%

According to The Botanical Records of China, *Gastrodia elata* often grows to a height of 1–2 m. The medicinal part of the rhizome is ellipsoid to nearly dumbbell shaped, with the length of 8–12 cm, 3–7 cm in diameter. Usually, the single fruit weighs 0.5–1 kg with denser nodes, which are covered with many triangular broadly ovate sheaths (Zhengyi, 1999).

## 3 Phytochemical overview

Since the 1950s, the metabolites contained in Rhizoma Gastrodiae have been widely studied. Previous reviews have also reported metabolites from Rhizoma Gastrodiae (Zhan et al., 2016; Guo et al., 2015). Other researchers classified the known chemical composition of Rhizoma Gastrodiae, mainly divided into aromatic metabolites, steroids, organic acids and esters, sugars and their glycosides, and other classes, based on the molecular structure of the parent nucleus of themetabolite (Yu, 2022). Selected bioactive metabolites from Rhizoma Gastrodiae are summarized in Table 2.

**TABLE 2:** Partial extract metabolites of GE.

Classification	Chemical metabolite	Ref
Aromatic metabolites	Gastrodin	Zuowei (2017)
	4-hydroxybenzyl methyl ether	Lee et al. (2007)
	4-hydroxybenzyl alcohol	Commission ChP (2020)
	4,4′-methylene biphenol	Chen et al. (2019b), Chen et al. (2019c)
	1-Furan-2-yl-2-(4-hydroxy-phenyl)-ethanone	Lee et al. (2007)
	bis-(4-hydroxybenzyl)sulfide	Shi et al. (2023)
	bis (4-benzyl) ether monobeta-D-galactopyranoside	Farooq et al. (2019)
	4,4,-sulfonyldiphenol	Zhan et al. (2016)
	5-(4-Hydroxybenzyloxymethyl)-2-carbaldehyde	Zhan et al. (2016)
	Gastropolybenzylol G	Chen et al. (2019b), Chen et al. (2019c)
	Gastropolybenzylol H	Chen et al. (2019b)
	4-hydroxybenzaldehyde	Ha et al. (2001)
	4-hydroxy-3-methoxybenzaldehyde	Ha et al. (2001)
	3,4-dihydroxybenzaldehyde	Lee et al. (2007)
	vanillin	Kim et al. (2019)
	N^6^-(4-hydroxybenzyl) adenine riboside	Nepal et al. (2019)
	Parish A-W	Yu (2022)
	1-O-(4-hydroxymethylphenoxy)-2-O-trans-cinnamoyl-b-D-glucoside	Wang et al. (2019)
	1-O-(4-hydroxymethylphenoxy)-3-O-trans-cinnamoyl-b-D-glucoside	Wang et al. (2019)
	1-O-(4-hydroxymethylphenoxy)-4-O-trans-cinnamoyl-b-D-glucoside	Wang et al. (2019)
	1-O-(4-hydroxymethylphenoxy)-6-O-trans-cinnamoyl-b-D-glucoside	Wang et al. (2019)
	para-hydroxybenzaldehyde	Li et al. (2019)
	(2)-γ-L-Glutamyl-L-[S-(4-hydroxybenzyl)]cysteinylglycine	Guo et al. (2015)
	Methyl (2)-γ-L-glutamyl-L-[S-(4-hydroxybenzyl)]cysteinylglycinate	Guo et al. (2015)
	(一)-(S_s_)-γ-L-Glutamyl-L-[S-(4-hydroxybenzyl)] cysteinylglycine sulfoxide	Guo et al. (2015)
	Ethyl (一)-(S_s_)-γ-L-glutamyl-L-[S-(4-hydroxybenzyl)] cysteinylglycinate sulfoxide	Guo et al. (2015)
	(一)-(R_s_)-γ-L-Glutamyl-L-[S-(4-hydroxybenzyl)] cysteinylglycine sulfoxide	Guo et al. (2015)
	Ethyl (一)-(R_s_)-γ-L-glutamyl-L-[S-(4-hydroxybenzyl)] cysteinylglycinate sulfoxide	Guo et al. (2015)
	(2)-γ-L-[N-(4-Hydroxybenzyl)]glutamyl-L-[S-(4-hydroxybenzyl)]cysteinylglycine	Guo et al. (2015)
	(p)-L-[S-(4-Hydroxybenzyl)]cysteinylglycine	Guo et al. (2015)
	4,4′-Dihydroxybenzyl sulfone	Pyo et al. (2004)
	4-Hydroxybenzylmethylether	Pyo et al. (2004)
	4,4-dihydroxy-dibenzyl ether	Pyo et al. (2004)
	4,4′-Dihydroxybenzyl sulfoxide	Pyo et al. (2004)
	4-[4'-(4″-Hydroxybenzyloxy) benzyloxy] benzyl methyl ether	Pyo et al. (2004)
	4,4′-Dihydroxy-diphenyl methane	Pyo et al. (2004)
	4,4′-Dihydroxy-dibenzylether	Pyo et al. (2004)
	5-Hydroxymethyl-2-furancarboxaldehyde	Pyo et al. (2004)
	Cirsiumaldehyde	Pyo et al. (2004)
	4-(4′-hydroxybenzyl) phenyl glucoside	Zhang et al. (2013)
	1′-hydroxymethyl-phenyl 4-hydroxy-3-(4″-hydroxybenzyl) benzyl ether	Zhang et al. (2013)
	bisphenol F	Huang et al. (2019)
Organic acids and their esters	citric acids	Zeng et al. (2023)
	1,5-dimethyl citrate	Lai et al. (2017)
	Docosanoic acid oxiranylmethyl ester	Yu (2022)
	6-methyl citrate	Yu (2022)
sterols	β-steroidal	Zhan et al. (2016)
	β-sitosterol glucoside	Zhan et al. (2016)
	3-O-(4′-hydroxybenzyl)-β-sitosterol	Yun-Choi et al. (1998)
	(3β,5α,6β)-Stigmastane-3,5,6-triol	Yu (2022)
	Stigmasta-3,5-diene	Yu (2022)
	Calcifediol	Yu (2022)
Saccharides	water-soluble polysaccharide (WGEW)	Dai et al. (2021)
	sulfated polysaccharide (WSS25)	Chen et al. (2015)
other	adenosine	Guan et al. (2017)

### 3.1 Aromatic metabolites

**FIGURE 2:**
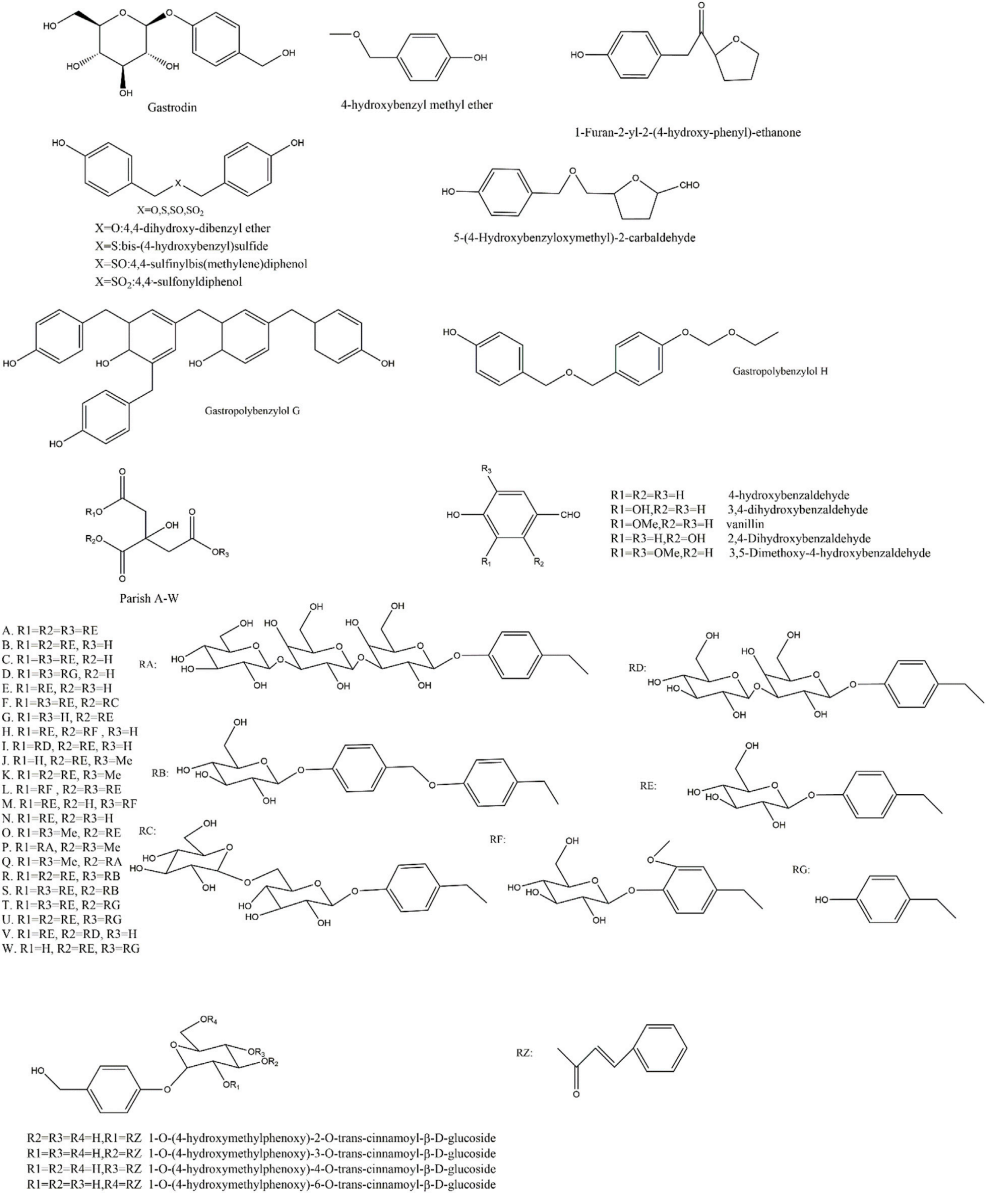
Structures of aromatic metabolites of GE.

The Aromatic metabolites in Rhizoma Gastrodiae are characterized by at least one benzene ring structure with a delocalized bond. 143 metabolites have been identified to date and there are still a lot to be discovered. Some researchers predicted that there are at least 189 possible parishin-like metabolites in gastrodia. Some researchers have predicted that at least 189 possible parishin-like metabolites are contained in gastrodia by offline two-dimensional liquid-liquid mass spectrometry and graphical similarity comparisons (Yu, 2022; Zhu et al., 2021). Some of the aromatic metabolites of Rhizoma Gastrodiae are shown in Figure 2.

Aromatic metabolites in Rhizoma Gastrodiae play a major role in disease treatment, especially the small molecule monobenzyl analogues. Gastrodin is the most important aromatic molecule in Rhizoma Gastrodiae because of its remarkable pesticide effect. This series of metabolites and their derivatives have a wide range of pharmacological effects either. Among them, Gastrodin and 4-hydroxybenzyl alcohol have stronger ability in eliminating ROS, avoiding oxidative damage to cells and improving the lifespan of the cells (Wang P. H. et al., 2016; He et al., 2021; Zhang et al., 2018; Lai, 2022). In addition, 4-hydroxybenzaldehyde and 4-hydroxy-3-methoxybenzaldehyde in Rhizoma Gastrodiae can effectively inhibit the activity of GABA transaminase, and aldehyde and hydroxyl groups are necessary groups for inhibiting GABA transaminase activity (Ha et al., 2001); The parishin analogues, including parishin A-W, cannot cross the blood-brain barrier to exert central effects due to their high molecular weight, but the hydrolysate p-hydroxybenzyl alcohol is capable of exerting central neuroprotective effects (Lai, 2022). Polybenzyl ethers are metabolites consisting of more than 2 benzyl units linked by oxygen atoms. Gastropolybenzylol H can activate MT1 and MT2 receptors in HEK293 cells, in which MT1 receptor is closely related to the function of the cardiovascular system. It can relax coronary arteries by agonising coronary β2 receptor, which have a certain protective effect on the cardiovascular system. On the other hand, activation of MT1 can alleviate brain inflammation and oxidative damage (Hardeland, 2021; Chen et al., 2019b). In addition, 4,4-dihydroxy-dibenzyl ether in polybenzyl ether showed some anti-platelet aggregation effects (Pyo et al., 2004).

Gastropolybenzylol G, 4,4′-methylene biphenol, as one of the polybenzyl metabolites, is able to activate MT1 and MT2 receptors in HEK293 cells, and the two p-hydroxy groups in methylene biphenols, are the key pharmacophore for the activation of MT1 and MT2 receptors (Chen et al., 2019b; Chen et al., 2019c). Moreover, MT1 is involved in the regulation of circadian rhythms, and activating MT1 can improve sleep. After MT2 receptor being activated, the expression of neurotrophic factor mRNA in the hippocampus region increased, the number of mitochondria increased, and nerve growth was promoted, which lead to improving cognition and anti-depression. Some of the metabolites have neuroprotective, anti-inflammatory and antioxidant effects (Zhang et al., 2013; Huang et al., 2019). Heteroatom aromatic metabolites formed by linking heteroatoms to each other had activities of anti-inflammatory, apoptosis-inhibiting, and neuroleptic-reducing. The same could be found when the benzyl unit was replaced by the alcohol hydroxyl group (Guo et al., 2015; Wang et al., 2019). In addition, It showed significant inhibition on topoisomerase I and II when the aromatic benzene ring connected to furan ring through the carbon chain or the oxygen atom, but indicated no obvious damage to HT-29, MCF-7, HEPPG 2 cells (Lee et al., 2007).

**FIGURE 3:**
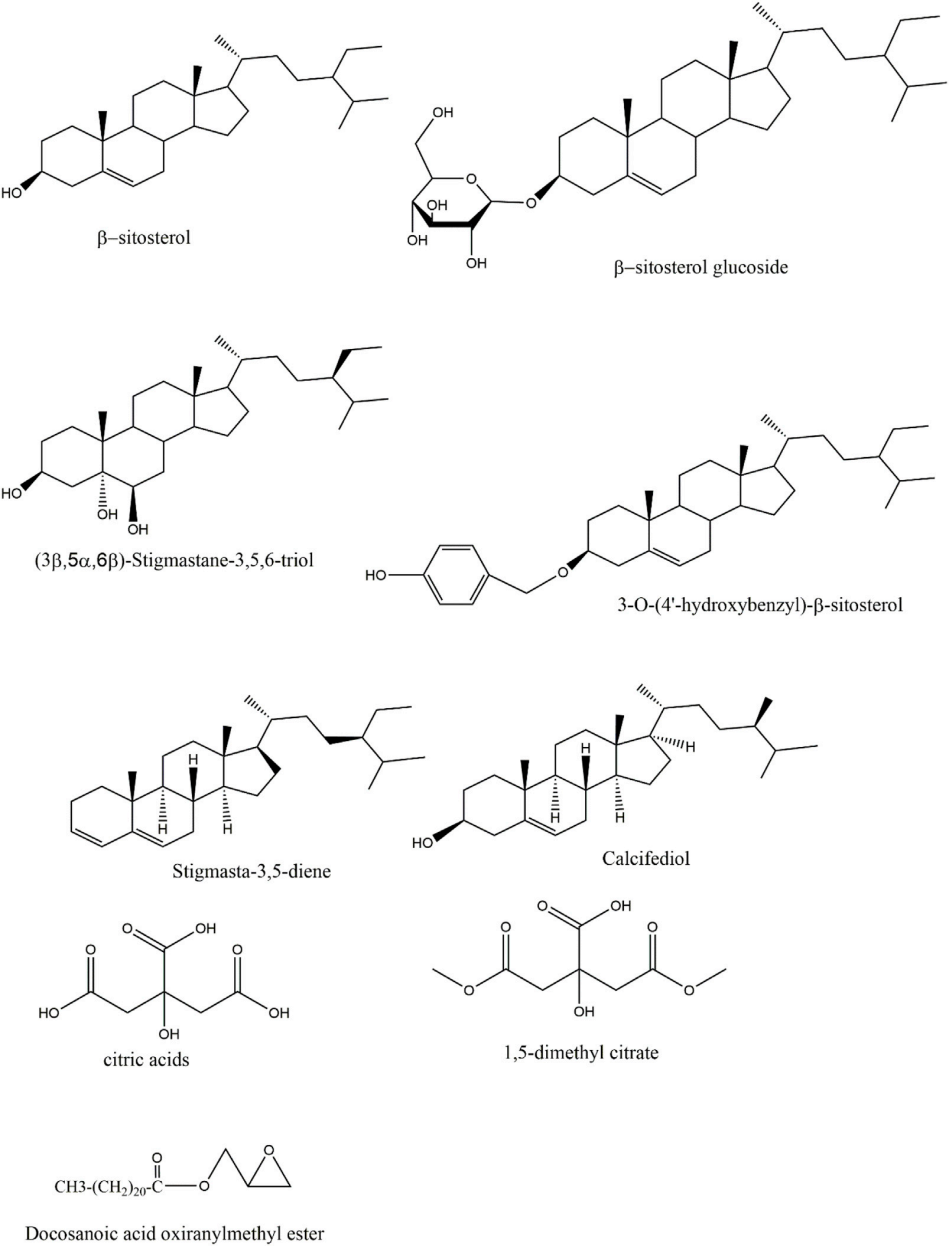
Structures of sterols and organic acids in GE.

The steroidal metabolites in Rhizoma Gastrodiae are featuring a perhydrocyclopentanophenanthrene skeleton with two angular methyl groups and a C-17 side chain. Few studies have been conducted on the steroid in Rhizoma Gastrodiae. Steroid metabolites in Rhizoma Gastrodiae that have been found include 3-O-(4′-hydroxybenzyl)-beta-sitosterol, β-sitosterol, β-sitosterol glucoside, 3β,5α,6β-Trihydroxystigmastane, stigmasta-3,5-diene, calcifediol and so on (Zhan et al., 2016; Wu et al., 2023). The related structures are shown in Figure 3. Among them, β-sitosterol has been proved to having a wide range of biological activities, such as anti-anxiety, sedation, analgesia, immune regulation, antibacterial, anti-cancer, anti-inflammatory, lipid-lowering, liver protection, heart protection, anti-oxidant, anti-diabetic activity (Babu et al., 2020; Khan et al., 2022). Most of the other steroids metabolites in Rhizoma Gastrodiae were also able to penetrate though the blood-brain barrier and demonstrated certain central and peripheral anti-inflammatory effects. Based on this evidence, it can be inferred that steroidal metabolites may also be a vital type of metabolites in Rhizoma Gastrodiae, which is responsible for the medicinal efficacy.

### 3.2 Organic acids and their esters of Rhizoma Gastrodiae

Themetabolites such as Docosanoic acid oxiranylmethyl ester, 6-methyl citrate, 1,5-dimethyl citrate, and citric acids have been isolated from GE (Zhan et al., 2016; Lai et al., 2017), and the related structures are shown in Figure 3. A study had confirmed that citric acid being given to mice ig could reduce brain inflammation and oxidative damage, while also exhibited certain protective effects on the liver (Abdel-Salam et al., 2014).

### 3.3 Saccharides and glycosides

Rhizoma Gastrodiae polysaccharides often has side chains formed by benzyl, which is one of the most important active metabolites. It has pharmacological effects such as regulating immunity, anti-cardiovascular and cerebrovascular diseases, anti-tumor, regulating intestinal flora, and improving osteoporosis (Chen et al., 2016; Zhao et al., 2022; Kim et al., 2012; Chen et al., 2012; Chen et al., 2015). Among them, the sulfated derivatives of water-soluble polysaccharide extracted from gastrodia polysaccharides (WGEW) are essential groups for inhibiting angiogenesis. It has been verified that the optimal degree of sulfation is between 0.173 and 0.194 (Chen et al., 2012). It was found that Rhizoma Gastrodiae polysaccharides with spherical conformations and dense structures are more effective in inducing late apoptosis of MCF-7 cells, thereby exerting anti-tumor effects after the analysis via using asymmetric flow field-flow fractionation (AF4), multi-angle light scattering (MALS), and differential refractive index (dRI) detectors in combination (AF4-MALS-dRI) (Dai et al., 2021).

Other categories Here, primarily composed of amino acids, nucleotides, and other metabolites that do not fit into the aforementioned categories. Rhizoma Gastrodiae contains a diverse array of amino acids and nucleotides. Pyroglutamic acid exhibits immunomodulatory effects, while adenosine demonstrates certain antiviral properties (Zhan et al., 2016).

In addition to the aforementioned five types of metabolites, Rhizoma Gastrodiae also contains trace elements such as La (Lanthanum), Sr (Strontium), and Zn (Zinc), which can serve as nutritional supplements for deficiencies associated with cardiovascular and cerebrovascular diseases (Xu and Xu, 2009).

## 4 Pharmacological effects of Rhizoma Gastrodiae

**FIGURE 4:**
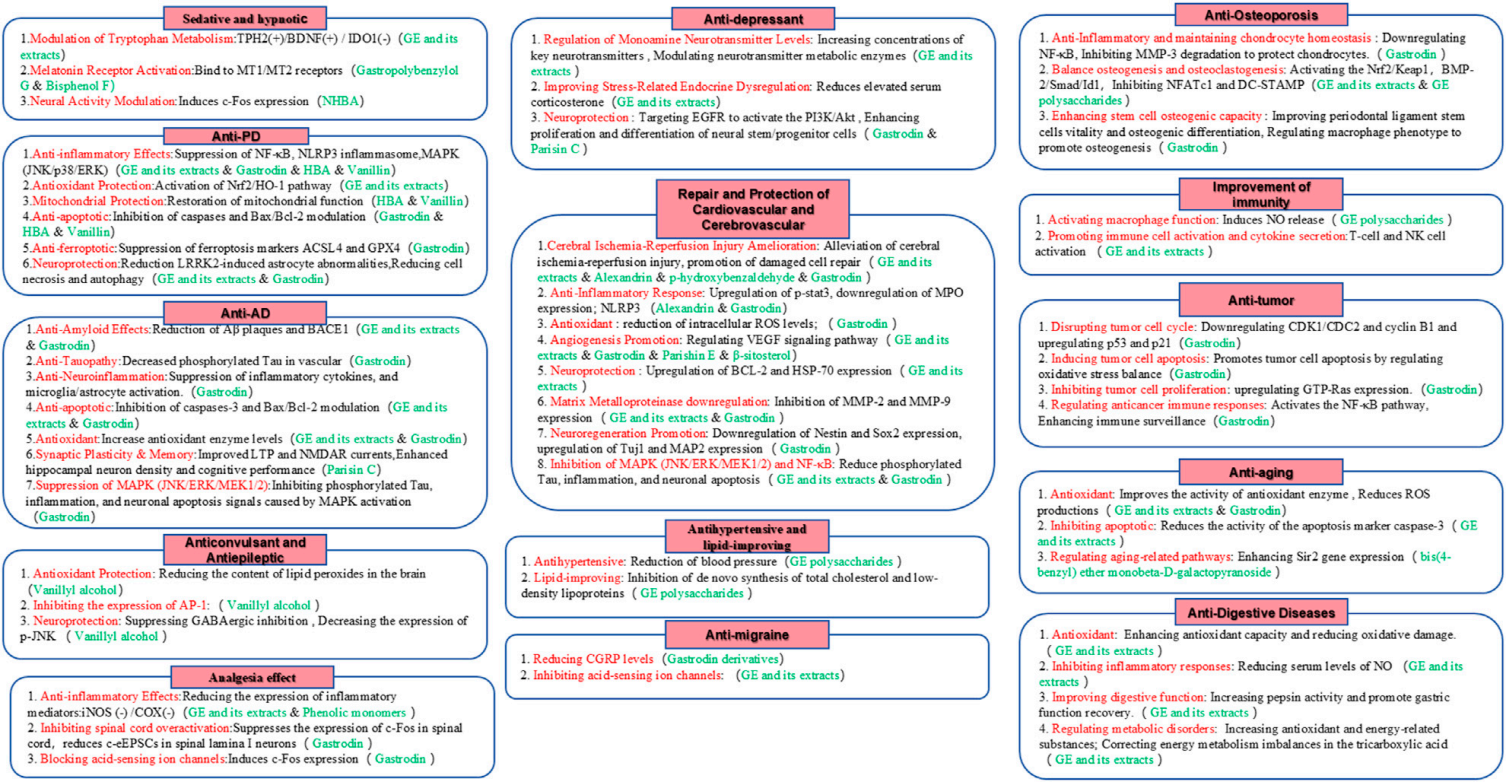
Pharmacological effects of Rhizoma Gastrodiae.

The bioactive metabolites of Rhizoma Gastrodiae are complex and include aromatic metabolites, carbohydrates and glycosides, volatile oils, proteins, organic acids and their esters, amino acids, vitamins, and trace elements. However, the most extensively studied metabolites of Rhizoma Gastrodiae are aromatic metabolites and plant polysaccharides (Heese, 2020; Zhu et al., 2019). These metabolites can enhance and protect the functions of the central nervous system, cardiovascular system, skeletal system, digestive system, endocrine system, urinary system, and respiratory system. Additionally, they exhibit a wide range of pharmacological effects, including boosting immunity, anti-tumor activities, antimicrobial properties, and delaying aging (Wu et al., 2023; Sun et al., 2023; Schloss et al., 2021). Detailed information is shown in Figure 4.

### 4.1 The pharmacological effects of Rhizoma Gastrodiae

The pharmacological effects of Rhizoma Gastrodiae on the nervous system include sedative and hypnotic effects, anti-Parkinson’s effects, anti-Alzheimer’s effects, antipsychotic effects, anticonvulsant effects, anti-vertigo effects, anti-epileptic effects, anti-stroke effects, and analgesic effects (Liu et al., 2018; Chen and Sheen, 2011; Schloss et al., 2021).

#### 4.1.1 Sedative and hypnotic effects

With increasing stress, insomnia has become a common condition in modern society. Its main triggers include disruptions in the biological clock caused by irregular daily routines, imbalances in sleep-related neurotransmitters, and dysfunction of the hypothalamic-pituitary-adrenal (HPA) axis (de Feijter et al., 2022). Research has shown that Gastrodin, p-hydroxybenzyl alcohol, and Parishin A can promote sleep by upregulating the sleep-related neurotransmitters 5-HT and GABA, while inhibiting the wake-promoting neurotransmitter DA (Zhou et al., 2024). Oral administration of fresh Rhizoma Gastrodiae powder and ethanol-steamed GE powder to C57 mice can reduce IDO 1, increase TPH 2 and BDNF levels, and elevate tryptophan levels, thereby enhancing the production of 5-HT and melatonin. This, in turn, improves the abundance of sleep-related neurotransmitters, promoting sedative, calming, and hypnotic effects in mice (Cheng et al., 2023). One of the active metabolites in GE, Gastropolybenzylol G, can activate melatonin receptors MT1 and MT2 in HEK293 cells, thereby further promoting sleep (Chen et al., 2019b; Chen et al., 2019c). Rhizoma Gastrodiae extract N^6^-(4-hydroxybenzyl) adenine riboside (NHBA) increases the expression of the proto-oncogene protein c-Fos in the ventrolateral preoptic area GABAergic neurons. Studies have confirmed that the expression of c-Fos significantly increases during rapid eye movement (REM) sleep, suggesting that NHBA activates the sleep center in the anterior hypothalamus to promote sleep. Some studies have also shown that gastrodin and p-hydroxybenzyl alcohol can improve sleep by affecting the HPA axis (Zhang et al., 2012). Detailed information is shown in Table 3.

**TABLE 3:** Sedative-hypnotic effect of Rhizoma Gastrodiae and its extracts.

Extract/metabolite	Inducer	Mode	Effects	Ref
Fresh GE powder	Chronic restraint stress	C57	IDO1↓ TPH2↑ BDNF↑	Cheng et al. (2023)
Ethanol-steamed GE powder	Chronic restraint stress	C57	IDO1↓ TPH2↑ BDNF↑	Cheng et al. (2023)
Gastropolybenzylol G	—	HEK293	MT1/2 (+)	Chen et al. (2019b)
Bisphenol F	—	HEK293	MT1/2 (+)	Chen et al. (2019c)
NHBA	—	Male ICR mice	c-Fos (+)	Zhang et al. (2012)

#### 4.1.2 Anti-Parkinson effect

Parkinson’s disease is a neurodegenerative disorder characterized by the death of dopaminergic neurons, leading to dopamine deficiency in the brain. The specific pathogenesis remains unclear, but its main features include mitochondrial dysfunction and increased reactive oxygen species (ROS), abnormal folding and aggregation of α-synuclein in synapses, elevated levels of pro-inflammatory factors in the brain microenvironment, ferroptosis, and the presence of pathogenic genes associated with Parkinson’s disease (Lu et al., 2022).

Studies on rats with brain injury have shown that gastrodin can modulate the NLRP3 signaling pathway, reduce the levels of ASC, TNF-α, IL-6, IL-1β, and IL-18, thereby alleviating inflammation and decreasing astrocyte accumulation. It promotes an increase in Bcl-2 and a decrease in Bax, and it also reduces Beclin-1, LC3-II, and P62 levels to prevent astrocyte apoptosis (Yang et al., 2022; Ng et al., 2016; Wang X. S. et al., 2016). Vanillin also possesses good antioxidant and anti-inflammatory properties. It can cross the blood-brain barrier (Salau et al., 2020), reducing the expression of p-JNK, p-P38, and p-ERK in rotenone induced human SH-SY5Y cells, thereby improving mitochondrial dysfunction, oxidative stress, and apoptotic cascades. It can inhibit cellular inflammation by suppressing the elevation of LPS induced ERK1/2, p38, NF-κB p65, JNK, IL-1β, IL-6, iNOS, and COX-2 (Dhanalakshmi et al., 2015; Yan et al., 2017; Kim et al., 2019).

Rhizoma Gastrodiae ethanol extract can inhibit TNF-α-induced vascular inflammation in HUVEC cells by suppressing oxidative stress and NF-κB activation. This is demonstrated by the reduced mRNA expression of ICAM-1, VCAM-1, E-selectin, macrophage chemoattractant protein-1 (MCP-1), and interleukin-8 (IL-8), showcasing its anti-inflammatory and anti-ROS effects (Hwang et al., 2009). Another report suggests that 0.1% Rhizoma Gastrodiae water extract can counteract the upregulation of Smad2/3 signaling caused by LRRK2 overactivation in Parkinson’s fruit flies with the LRRK2-G2019S mutation (the most common familial Parkinson’s disease mutation) by activating the Nrf2 signaling pathway. This restores normal microglial function and improves their motor condition,. In individuals with Parkinson’s, where abnormal folding and aggregation of α-Syn are typically observed, Lrrk2 overactivation is often found. It is *therefore* hy*p*othesized that the Rhizoma Gastrodiae water extract may improve the Parkinson’s condition by alleviating the abnormal folding and aggregation of α-Syn, thereby protecting neurons (Lin et al., 2021). HBA can induce the increased expression of antioxidant enzymes in SH-SY5Y cells, thereby reducing oxidative stress, exerting mitochondrial protection, and inhibiting apoptosis. It inhibits the ROS-dependent JNK/Jun/caspase-3 signaling pathway, effectively protecting dopaminergic neurons, reducing oxidative damage and death of neurons, and improving Parkinson’s symptoms (Lai, 2022).

**FIGURE 5:**
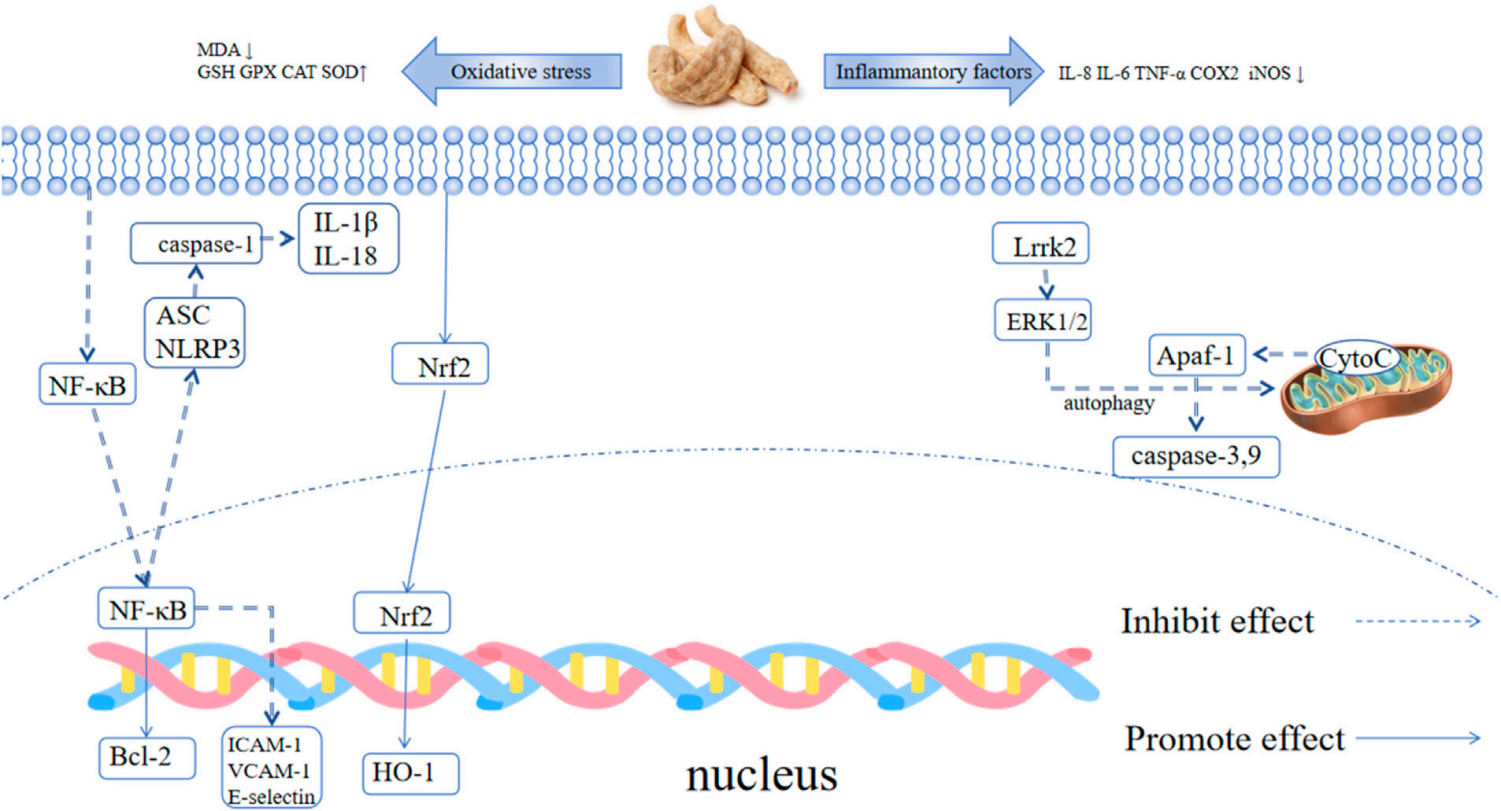
Mechanism of action of GE in Parkinson’s disease.

H_2_O_2_ treatment downregulated the protein expression of Nrf2, HO-1, GPX4 and FPN1 in rat C6 cells, while pretreatment with 25 μM gastrodin significantly reversed this trend (the protein expression of Nrf2, HO-1 and GPX4 was 1.8-fold, 2.1-fold and 1.5-fold higher than that in the H_2_O_2_ group, respectively, *p* < 0.01). In addition, gastrodin inhibited the H_2_O_2_-induced upregulation of ACSL4 and COX2 protein expression (the expression of ACSL4 and COX2 in the 25 μM gastrodin group was approximately 30% lower than that in the H_2_O_2_ group, *p* < 0.05), and reduced intracellular iron accumulation (the intracellular iron concentration in the 25 μM gastrodin group was approximately 25% lower than that in the H_2_O_2_ group, *p* < 0.01) (Jiang et al., 2020). Gastrodin can regulate neurotransmitters, exhibit both antioxidant and anti-inflammatory properties, inhibit the activation of microglial cells, manage mitochondrial cascade reactions, and enhance neurotrophic factor levels (Liu et al., 2018). Table 4 summarizes the effects of GE and its extracts on Parkinson’s disease, while Figure 5 illustrates the mechanism of action of GE.

**TABLE 4:** The role of Rhizoma Gastrodiae and its extracts in Parkinson’s disease.

Extract/metabolite	Mode (animal/cell)	Mechanism and effects	Ref
99% ethanol Extract of Gastrodia elata Blume	TNF-α induced HUVEC	ICAM-1↓ VCAM-1↓ IL-8↓ E-selectin↓ MCP-1↓	Hwang et al. (2009)
Water extraction of Gastrodia elata Blume	LRRK2-G2019S *Drosophila* LRRK2-G2019S Mouse	Nrf2(+) Lrrk2 (−) HO-1↑ Smad2/3 (−)	Lin et al. (2021)
	TBI SD female rats	astrocytes accumulation↓ IL-6↓ rotarod performance↑ TNF-α↓	Ng et al. (2016)
Gastrodin	TBI male rats	NLRP3↓ ASC↓ TNF-α↓ Caspase-1↓ caspase-11↓ IL-1β↓ IL-18↓	Yang et al. (2022)
	LPS induces C57BL/6 mice	Bcl-2↑ Bax↓ LC3-II/I ratio↓	Wang et al. (2016b)
	primary astrocyte	Beclin-1↓ P62↓ Necrosis cell↓ Autophagic cell↓	
	H2O2 induces C6	ACSL4↓ COX2↓ FPN1↑ MDA↓ GSH↑ GPX4↑ Nrf2↑ HO-1↑	Jiang et al. (2020)
HBA	6-OHDA induces SH-SY5Y	SOD↑ GSH-Px↑ CAT↑ Apaf-1↓ MMP↑ ADP/ATP↓Caspase-3,9↓ Mitochondria cytochrome p-pJNK/pJNK↓ p-c-Jun/c-Jun↓	Lai (2022)
Vanillin	Rotenone induces SH-SY5Y	Mitochondria membrane potential↑	Dhanalakshmi et al. (2015), Yan et al. (2017), Kim et al. (2019)
	LPS induces BV-2	Bcl-2/Bax↑ Caspase-3,8,9↓	
	LPS induces Wistar rats	Mitochondria cytochrome c↑ iNOS↓ COX-2↓ IL-1β↓ IL-6↓ TNF-α↓Cytosolic cytochrome c↓ NF-κB p65↓ p-JNK↓ p-p38↓ p-ERK↓	

#### 4.1.3 Anti-Alzheimer’s disease (AD)

Alzheimer’s disease is an age-related neurodegenerative disorder, with 95% of cases being non-hereditary sporadic cases. Its pathological mechanisms are complex, with major hypotheses including β-amyloid (Aβ) deposition, neuroinflammation, excessive phosphorylation of tau proteins, and synaptic neuronal loss (Jiang et al., 2023). It is specifically manifested as cognitive impairment and memory decline (Lindsay et al., 2002; Sulistio and Heese, 2016).

The water extract of Rhizoma Gastrodiae (WGE) significantly ameliorates the pathological conditions in Aβ-induced Alzheimer’s disease model *Drosophila*. In the lifespan assay, treatment with 5 mg/g WGE extended the median lifespan of Aβ42 *Drosophila* by 7 days (26.9% prolongation) and the maximum lifespan by 7 days (both *P* < 0.001). Climbing experiments showed that 5 mg/g WGE enhanced the climbing ability of *Drosophila* by 14.4% (*P* < 0.001), 11.6% (*P* < 0.01), and 9.74% (*P* < 0.05) on days 12, 19, and 23, respectively. In retinal degeneration assays, WGE (5 mg/g) increased the number of rhabdomeres per ommatidium by 0.97, comparable to the effect of 10 μmol/g donepezil (*P* < 0.001). *In vitro* experiments further confirmed that WGE alleviates Aβ-induced apoptosis in PC12 cells by enhancing the activities of antioxidant enzymes such as CAT, SOD, and GPX (increased to 120%, 150%, and 160%, respectively), reducing ROS production (decreased to 80%), and inhibiting Caspase-3 activity (31.8% reduction). Moreover, 1000 μg/mL GE completely reverses Aβ-induced cytotoxicity (Lin et al., 2021; Ng et al., 2013). Rhizoma Gastrodiae extract improved the viability of Aβ-treated PC12 cells in a dose-dependent manner, attenuating Aβ-induced oxidative and apoptotic stress. Rhizoma Gastrodiae also significantly upregulated the enzymatic activities of catalase, superoxide dismutase, and glutathione peroxidase, leading to a reduction in reactive oxygen species production and the activity of the apoptotic marker caspase-3 (Ng et al., 2013). It also alleviate vascular cognitive impairment by increasing the acetylcholine content and stabilizing the structure and function of mitochondria (Li et al., 2018).

There is also evidence indicating that Gastrodin can improve memory in mice, reduce the deposition of Aβ amyloid plaques, the number of astrocytes and activated microglial cells, and decrease the expression of TNF-α and IL-1β in N9 cells. At the same time, it regulates the TLR4/TRAF6/NF-κB pathway to alleviate neuroinflammation and microglial activation in the AD model, thereby exerting anti-AD effects (He et al., 2021; Hu et al., 2014). It can also downregulate the expression of amyloid precursor protein (APP) cleaving enzymes to inhibit the accumulation of Aβ and the abnormal phosphorylation of Tau protein, improve the neurofibrillary tangles (NFTs) caused by abnormal Tau phosphorylation, and drive the non-amyloidogenic pathway to prevent Alzheimer’s disease (AD) (Shi et al., 2020; Li and Qian, 2016; Zhang et al., 2016). The extract of Rhizoma Gastrodiae, Parisin C, can inhibit the abnormal activation of N-methyl-D-aspartate receptors (NMDAR) in the Wistar rat AD model induced by Aβ1-42, thereby exerting anti-AD effects.

Intracerebroventricular (i.c.v.) injection of Aβ1-42 oligomers (2 μmol/L) significantly inhibited NMDAR-dependent long-term potentiation (LTP) in the hippocampal dentate gyrus of rats, reducing the LTP amplitude from 195.1% ± 9.6% in the control group to 148.7% ± 6.5% (*P* < 0.05). Pretreatment with Parishin C (20 mg/kg, intraperitoneal injection) restored the LTP amplitude to 179.0% ± 8.4% (P < 0.05), while i. c.v. administration of 10 μmol/L Parishin C further increased the LTP amplitude to 210.2% ± 22.1% (*P* < 0.05). Electrophysiological experiments further confirmed that Aβ1-42 oligomers (2 μmol/L) reduced the NMDAR current in hippocampal neurons to 71.0% ± 5.0% of the pre-administration level (*P* < 0.05), and the current continued to decrease to 44.1% ± 7.1% after drug washout. Pretreatment with Parishin C (10 μmol/L) significantly antagonized this inhibitory effect (*P* < 0.05). Abnormal activation of NMDAR may lead to neuronal hyperexcitability and disrupt neuroprotective mechanisms. Aβ1-42-induced inhibition of NMDAR function impairs synaptic plasticity of hippocampal neurons. By specifically protecting NMDAR current, Parishin C improves Aβ-induced hippocampal neuronal injury, thereby protecting cells, maintaining information processing ability, and ultimately improving advanced cognitive functions such as learning and memory (Liu Z. et al., 2016). The relevant information is shown in Table 5.

**TABLE 5:** The effects of Rhizoma Gastrodiae and its extracts on AD.

Extract/metabolite	Mode (animal/cell)	Mechanism and effects	Ref
Water extraction of GE	Aβ-Transgenic Drosophila	Locomotor ability↑ lifespan↑	Ng et al. (2013)
	Aβ-treated P12	Degenerating rhabdomeres↓apoptotic↓ SOD↑ CAT↑ GPx↑caspase-3↓	
Gastrodin	Tg2576 mice	Memory↑ Aβ plaques burden↓ Microglia and astrocytes ability↓ IL-1β↓ TNF-α↓	Hu et al. (2014)
	2-VO-Vascular dementia rat	Density of hippocampal neurons↑ neuronal alignment Memory↑ p-Tau↓	Shi et al. (2020)
	Aβ1–42 induces C57BL/6	IL-1β↓ TNF-α↓ IL-6↓ iNOS↓	Li and Qian (2016)
	Primary NPCs	Bcl-2↑ Caspase-3↓ Bax↓ Bcl-XL↑ p-JNK↓ p-ERK↓ p-MEK1/2↓	
	Tg2576 C57BL/6-SJL	Mouse memory↑ SOD↑ CAT↑	Zhang et al. (2016)
	H2O2 induces SH-SY5Y	MDA↓ pPKRThr446↓ peIF2aSer51↓ BACE1↓	
Parisin C	Aβ1-42 induces wister rats	Suppression of LTP↓ NMDAR currents↑	Liu et al. (2016a)

#### 4.1.4 Anticonvulsant and antiepileptic effects

Kainic acid is a neurotoxic substance that acts as an agonist for glutamate receptors in the central nervous system to increase oxidative stress and neuronal damage. It can be used for establishing rat models of epilepsy (Chen and Sheen, 2011). Rhizoma Gastrodiae and *Uncaria rhynchophylla* used in combination for kainic acid (KA)-induced seizures in SD rats significantly delayed the onset time of wet dog shakes in rats compared to the use of *U. rhynchophylla* alone, suggesting that Rhizoma Gastrodiae has a certain anticonvulsant effect (Hsieh et al., 1999). Subsequent research confirmed that vanillyl alcohol in Rhizoma Gastrodiae can reduce seizures induced by ferric chloride in rats. It can relieve symptoms of wet dog-like shaking by reducing the content of lipid peroxides in the brain, inhibiting the expression of AP-1, suppressing GABAergic inhibition, and decreasing the expression of p-JNK (Hsieh et al., 2007).

#### 4.1.5 Analgesia effect

Rhizoma Gastrodiae has been applied in analgesia, yet the underlying mechanism of its pain-relieving effect remains poorly investigated. The ethanol extract of GE (GEE) and phenolic monomers can reduce the expression of iNOS and COX-2 in RAW264.7 cells and decrease the writhing times in mice. Additionally, GEE can reduce COX-I and COX-II in RBL 2H3 cells, thereby demonstrating a certain analgesic effect (Liu et al., 2018; Lee et al., 2006). In 2016, researchers confirmed that gastrodin inhibits the expression of c-Fos in the spinal cord of mice, as well as C-fiber evoked EPSCs (c-eEPSCs) in spinal lamina I neurons. It has an analgesic effect on peripheral inflammation induced spinal spontaneous pain, mechanical and thermal hypersensitivity induced pain. This hypersensitivity is not dependent on opioid receptors and does not develop tolerance (Qiu et al., 2014). The analgesic mechanism may function by partially blocking acid-sensing ion channels, thereby inhibiting the presynaptic enhancement effect in the spinal cord caused by inflammation (Qiu et al., 2014; Xiao et al., 2016).

#### 4.1.6 Antidepressant effects

Rhizoma Gastrodiae exhibits therapeutic efficacy not only in neurodegenerative diseases but also demonstrates specific effects on psychiatric disorders. After administering WGE to Sprague-Dawley (SD) rats, the concentration of 5-hydroxytryptamine (5-HT) in the prefrontal cortex and dopamine (DA) in the striatum significantly increased. This led to a reduction in immobility time during the Forced Swim Test (FST) for the rats. It also decreased the levels of 5-HT and serum corticosterone in rats subjected to the unpredictable chronic mild stress (UCMS) model and improved grooming behavior and activity levels (Huang et al., 2021). Moreover, WGE can exert antidepressant effects by reducing the activity of monoamine oxidase (MAO-A) in PC12 cells and increasing the activity of tyrosine hydroxylase (TH) (Chen et al., 2009; Lin et al., 2016). Metabolomics (UPLC-QTOF-MS) combined with transcriptomics, network pharmacology, and molecular docking have confirmed that gastrodin and Parishin C, the key bioactive metabolites of Gastrodia elata, target the epidermal growth factor receptor (EGFR), activate the PI3K/Akt signaling pathway, and promote the proliferation of hippocampal neural stem/progenitor cells (NSPCs, ∼30% increase in BrdU + cells vs. model group) and neuronal differentiation (∼25% increase in BrdU + NeuN + cells). These effects alleviate chronic mild stress (CMS)-induced depressive-like behaviors, as evidenced by a ∼20% increase in sucrose preference and a ∼25% reduction in immobility time in the tail suspension test. These findings suggest that bioactive factors such as gastrodin and Parishin C exert antidepressant effects via the “EGFR-PI3K/Akt” axis (Huang et al., 2021; Liu et al., 2025).

### 4.2 Effects of gastrodia on cardiovascular

Rhizoma Gastrodiae has cardiovascular effects, including repair and protection of the cardiovascular system (Yang et al., 2022; Zhu et al., 2018; Baral et al., 2015), improvement of dizziness symptoms (Liu et al., 2018), antihypertensive effects (Lee et al., 2012), relief of migraines and hemiplegia (Wang P. H. et al., 2016), and anti-arteriosclerosis effects (Lee et al., 2012; Kim et al., 2012). The effects of Rhizoma Gastrodiae and its extracts on the cardiovascular system are shown in Table 6.

**TABLE 6:** Rhizoma Gastrodiae and its extracts’ effects on the cardiovascular and cerebrovascular systems.

Extract/metabolite	Mode (animal/cell)	Disease	Mechanism and effects	Ref
Gastrodin	Rats	TBI	Alleviated neural injury TNF-α↓ IL-1β↓ IL-18↓ASC↓ GSDMD↓ caspase-1↓ caspase-11↓ NLRP3↓	Yang et al. (2022)
70% ethanol extraction of GE	NSCs	—	Neuronal differentiation of NSCs↑ Tuj1↑ MAP2↑ Nestin↓	Baral et al. (2015)
Acid polysaccharides Crude polysaccharides	Fed a high-fat diet SHR	Hypertension Hyperlipidemia	Lowering blood pressure TC↓ TG↓ LDL↓ HDL↑	Lee et al. (2012)
Gastrodin	I/R surgery rats	Cerebral I/R	Infarct volume↓ IL-1β↓ COX-2↓ CA1 region cellular edema and nuclear loss↓ iNOS↓ cleaved caspase-3↓	Liu et al. (2016b)
	Nitroglycerin induced	Migraine	NO↑ CGRP↓ c-Fos↓	Wang et al. (2016a)
Ethyl acetate of GE	I/R surgery SD rats	Cerebral I/R	Cerebral infarction rate↓ bcl-2↑ TUNEL-positive↓HSP-70↑ Neurological scores↓ Cerebral index↓	Luo et al. (2022)
Alexandrin	HT-22	—	Cell viability↑ p-STAT3/STAT3↑	Lee et al. (2009)
Gastrodin	HT-22	—	Cell viability↑ MMP-9↓	
Para-hydroxybenzaldehyde	HT-22	—	Cell viability↑ MPO↓	
Polysaccharide of GE	PTK787 fed zebrafish		ISVs↑	Lee et al. (2009)
Ethanol extract from rhizome of GE	TNF-α induces HUVEC	—	MMP-9↓ MMP-2↓	Dong et al. (2021)
Gastrodin	A/R H9c2	—	ROS↓ LDH↓ CPK↓ 14-3-3η↑	Zhu et al. (2018)
	Glucose-induced H9c2/HL-1	—	GSH↑ SOD↑ CAT↑ ROS↓	Li et al. (2019)

#### 4.2.1 Repair and protection of cardiovascular and cerebrovascular systems

Rhizoma Gastrodiae has been shown to exert potent therapeutic and protective effects against cardiovascular diseases. Emerging evidence from current studies suggests that its extracts, including alexandrin, para-hydroxybenzaldehyde, and gastrodin, are capable of ameliorating cerebral ischemia-reperfusion injury and facilitating repair of damaged cells (Luo et al., 2022; Li et al., 2019). These three types of metabolites can improve the viability of HT22 cells after oxygen-glucose deprivation/reperfusion (OGD/R) treatment. Western blot (WB) experiments also confirmed that alexandrin can upregulate the expression of p-stat3 and downregulate the expression of MPO, thereby alleviating oxidative stress and inflammatory responses in the cardiovascular system caused by abnormal MPO(myeloperoxidase) expression. In addition, Gastrodin downregulates the expression of MMP-9, which is a high-risk marker for cardiovascular diseases. Elevated levels of MMP-9 indicate a poor prognosis for cardiovascular diseases (Luo et al., 2022). The study on gastrodin ameliorating cerebral ischemia-reperfusion injury, despite verifying the expression changes of p-STAT3 and MPO via Western blot, lacked a positive control drug (such as edaravone, a commonly used neuroprotective agent), making it impossible to comparatively assess gastrodin’s therapeutic superiority. Moreover, the evaluation of brain injury severity relied solely on histopathological scoring without incorporating functional indicators like neurological deficit scores, thus failing to comprehensively reflect its therapeutic efficacy. Intraperitoneal injection of Gastrodin in TBI (Traumatic brain injury) rats can alleviate the reduction in the number of neurons, nuclear shrinkage, and degeneration in the brainstem area caused by TBI. It also reduces the expression of inflammatory factors TNF-α, IL-1β, and IL-18, and downregulates the expression of pyroptosis-related proteins GSDMD, NLRP3, ASC, caspase-1, and caspase-11. This suggests that Gastrodin may improve brain injury by inhibiting the NLRP3 inflammasome signaling pathway to affect pyroptosis (Yang et al., 2022). GE ethyl acetate extract also exhibits certain neuroprotective effects. Administering it to SD rats with ischemia-reperfusion injury can increase the expression of BCL-2 and HSP-70, enhance brain cell survival rate, and improve brain damage (Duan et al., 2015). However, this study did not clearly determine the content ratio of active components in the extract, making it impossible to ascertain whether a single component or multiple components synergistically contribute to the effects, thereby affecting the accuracy of mechanism analysis.

The 95% ethanol extract of GE has been demonstrated to promote angiogenesis in zebrafish models and exhibit protective effects against ischemic cardiovascular diseases and atherosclerosis when administered *in vivo* (Liu et al., 2020). Using metabolomics technology (LC-TOF-MS), combined with zebrafish models and grey correlation analysis, ten metabolites highly correlated with pro-angiogenic activity (correlation coefficient >0.9) were identified, including gastrodin, parishin E, β-sitosterol, etc. Experiments confirmed that the extract of Gastrodia elata showed the optimal pro-angiogenic effect at 100 μg/mL, significantly promoting the growth of intersegmental vessels in zebrafish. Network pharmacology analysis revealed that these metabolites exert their effects by targeting VEGFA, TNF and other targets, and regulating signaling pathways such as VEGF, MAPK, and NF-κB (Liu et al., 2020). Thus, GE demonstrates a positive effect in the therapeutic intervention and protective management of cardiovascular diseases.

Furthermore, experimental evidence has demonstrated that *in vitro* co-culture of neural stem cells (NSCs) with gastrodin leads to a significant downregulation of Nestin and Sox2 expression, accompanied by an upregulation of Tuj1 and MAP2. These findings suggest that gastrodin exhibits neuroregenerative properties in NSCs, facilitating the repair of brain neural injuries (Baral et al., 2015). Administration of gastrodin to A/R H9c2 cells can reduce intracellular ROS levels, decrease the release of LDH and CPK, and enhance the expression of 14-3-3η, thereby reducing cell apoptosis and exerting a protective effect on the cells (Zhu et al., 2018). The licorice saponin can also protect high glucose-induced H9c2 and HL-1 cardiomyocyte from toxicity, oxidative stress, and apoptosis by enhancing the nuclear translocation of Nrf2 mediated by GSK-3β, increasing GSH, SOD, and CAT levels, and reducing ROS. This suggests that licorice saponin may also be used as a potential treatment for diabetic cardiomyopathy (Dong et al., 2021).

#### 4.2.2 Reduce hypertension

Researchers have discovered that Rhizoma Gastrodiae acidic polysaccharides and crude polysaccharides can reduce hypertension in spontaneously hypertensive rats (SHR). These metabolites simultaneously increase high-density lipoprotein cholesterol (HDL-C) levels while lowering total cholesterol (TC) and low-density lipoprotein cholesterol (LDL-C) levels, . They also inhibit *de novo* synthesis of total cholesterol and low-density lipoproteins in rats, thereby improving hemorheology through multiple pathways and reducing the incidence of cardiovascular diseases and atherosclerosis (Lee et al., 2012; Kim et al., 2012).

#### 4.2.3 Anti-migraine

Calcitonin gene-related peptide (CGRP) is significantly elevated in migraines (Russo and Hay, 2023). Researchers have found that using gastrodin to synthesize gastrodin derivatives (Gastrodin-D) can reduce plasma CGRP levels in SD rats, exerting an anti-migraine effect (Wang P. H. et al., 2016). There are also reports indicating that Rhizoma Gastrodiae inhibits acid-sensing ion channels, blocks pain transmission, and alleviates migraines (Feng, 2022).

### 4.3 Other effects of Rhizoma Gastrodiae

Rhizoma Gastrodiae is commonly used for neurological disorders (Liu et al., 2018), brain injuries (Baral et al., 2015) and the treatment of cardiovascular diseases (Lee et al., 2012). However, the medical applications of Rhizoma Gastrodiae are not confined to the aforementioned uses. As ongoing research deepens the understanding of its pharmacological mechanisms, investigators have continued to uncover novel therapeutic effects of Rhizoma Gastrodiae, with osteoporosis treatment being a notable example (Chen et al., 2015), boosting immunity (Chen et al., 2016), treating gastritis (Lee et al., 2009), delaying aging, and anti-tumor effects (Farooq et al., 2019; Liang et al., 2017; Ahn et al., 2007).

#### 4.3.1 Reducing osteoporosis

Current studies indicate that both gastrodin and Rhizoma Gastrodiae polysaccharide WSS25 exhibit specific effects on osteoporosis. Gastrodin works by inhibiting the nuclear translocation of NF-κB in chondrocytes, downregulating the expression of TNF-α and IL-1β, and inhibiting MMP-3 degradation to maintain chondrocyte homeostasis. In a model of LPS-induced human periodontal ligament stem cells, Gastrodin can also mitigate the attack of inflammatory factors such as TNF-α on these cells, enhance the vitality and osteogenic capacity of human periodontal ligament stem cells, increase the M2/M1 ratio, and promote the differentiation and formation of osteoblasts. In the glucocorticoid-induced osteoporosis rat model, the use of Gastrodin was found to improve osteoporosis status by activating the Nrf2/Keap1 pathway and upregulating the expression of OCN, BMP-2, and RUNX2. Moreover, Gastrodin also ameliorates osteoporosis by blocking the formation, maturation, and migration of osteoclasts through the inhibition of the NFATc1 gene and specific transmembrane protein DC-STAMP (Chen et al., 2018).

WSS25 is a sulfated polysaccharide extracted from the rhizome of Rhizoma Gastrodiae. It binds to bone morphogenetic protein 2 (BMP-2) in hepatocellular carcinoma cells, and BMP-2 may simultaneously regulate both osteoclasts and osteoblasts. WSS25 effectively inhibits the expression of TRAP, NFATc1, MMP-9, and cathepsin K in RAW264.7 or BMMs cells induced by RANKL, thereby suppressing the formation and differentiation of osteoclasts and alleviating bone resorption. On the other hand, WSS25 promotes the expression of osteogenic markers such as OCN, BMP-2, and RUNX2, enhancing osteoblast differentiation and improving bone strength. Long-term administration of WSS25 significantly reduces bone loss in ovariectomized mice and mitigates the inhibition of the BMP-2/Smad/Id1 signaling pathway caused by the BMP-2 antagonist noggin. These findings suggest that Rhizoma Gastrodiae has potential applications in the treatment of osteoporosis, warranting further exploration (Chen et al., 2015).

#### 4.3.2 Improvement of immunity

Rhizoma Gastrodiae polysaccharide GDP has been shown to induce NO release in RAW264.7 cells, thereby activating immune cells and significantly enhancing the phagocytic activity of RAW264.7 macrophages (Chen et al., 2016). In tumor-bearing mice, administration of the Rhizoma Gastrodiae water extract has been shown to upregulate serum levels of IL-2 and IFN-γ, induce T-cell activation, and enhance immune responses. Additionally, separate studies have demonstrated that the silkie chicken Rhizoma Gastrodiae nutrient solution significantly increases thymus weight ratio in mice, promotes NK cell activation, and potently enhances immune system function (Li et al., 2019; Russo and Hay, 2023).

#### 4.3.3 Anti-tumour effect

Studies have demonstrated that Rhizoma Gastrodiae exhibits antitumor activity, with underlying mechanisms involving multiple aspects. Specifically, gastrodin treatment leads to an increase in the proportion of subG1 and G2/M phase cells, accompanied by a decrease in G0/G1 phase cells, in DBTRG-05MG glioma cells. This is associated with downregulation of CDK1 (cyclin-dependent kinase 1)/CDC2 and cyclin B1, as well as upregulation of p53 and p21, thereby disrupting the tumor cell cycle. Gastrodin increases intracellular ROS levels without raising mitochondrial ROS levels, decreases GSH levels in DBTRG-05MG cells, increases SOD levels, and reduces GPx and CAT levels, thereby promoting glioma cell apoptosis (Liang et al., 2017). Additionally, the ethyl ether extract of Rhizoma Gastrodiae inhibits the proliferation of B16 cells by upregulating GTP-Ras expression (Heo et al., 2007). Other studies have indicated that gastrodin, the main metabolite of Gastrodia elata, attenuates H22 tumor cell transplantation-induced decrease in CD4^+^ T cells. This is accompanied by a dose-dependent reduction in IFN-γ and IL-2 expression, inhibition of IL-4 upregulation, modulation of CD4^+^ T cell subpopulation ratios, and activation of the NF-κB pathway, thereby exerting an antitumor effect through anticancer immune responses (Shu et al., 2013).

#### 4.3.4 Anti-aging effect

In addition to its broad pharmacological effects, Rhizoma Gastrodiae and its extracts have been shown to possess remarkable anti-aging properties. Gastrodin can enhance the antioxidant capacity (SOD, CAT) of black fruit flies, extending their lifespan and improving their oxidative resistance (He et al., 2021). The aqueous extract of Rhizoma Gastrodiae significantly upregulates the enzyme activities of catalase, superoxide dismutase, and glutathione peroxidase, leading to the production of reactive oxygen species and a decrease in the activity of the apoptosis marker caspase-3, resulting in αβ-induced fruit flies having longer lifespans, better motor function, and fewer small eye degenerations. The mechanism of action may involve alleviating αβ-induced oxidative and apoptotic stress (Ng et al., 2013). A new metabolite, “bis (4-benzyl) ether monobeta-D-galactopyranoside”, ametabolite isolated from the rhizome of Gastrodia elata (GE), has been shown to significantly extend the lifespan of two yeast strains, K6001 and YOM36. Additionally, experimental evidence indicates that this metabolite reduces reactive oxygen species (ROS) and malondialdehyde (MDA) levels in BY4741 yeast cells while upregulating the expression of catalase (CAT) and thiol peroxidase (CPx). Moreover, in yeast strain K6001, the metabolite enhances Sir2 gene expression and inhibits the *U*th1/TOR signaling pathway, thereby promoting lifespan extension and exerting anti-aging effects (Farooq et al., 2019).

#### 4.3.5 Treatment of digestive diseases

GE extract has been demonstrated to mitigate oxidative stress in the stomach, enhance energy and amino acid metabolism, alleviate inflammatory responses, and thereby alleviate gastritis Using ^1^H NMR metabolomics, we intervened rats with chronic atrophic gastritis model with water extract (T1), n-butanol extract (T2), ethyl acetate extract (T3) and petroleum ether extract (T4) of Gastrodia elata continuously for 21 days. It was found that compared with the model group, T1 group significantly reduced the level of malondialdehyde (MDA) in gastric tissue (*p* < 0.001), significantly increased the activities of superoxide dismutase (SOD) and glutathione (GSH), decreased the levels of serum nitric oxide (NO) and xanthine oxidase (XOD), and increased pepsin activity by 28.5%. Metabolomic analysis showed that it could regulate 34 differential metabolites, including decreasing the levels of branched-chain amino acids such as leucine (*p* < 0.01), isoleucine and acetate, increasing antioxidant substances such as glucose (*p* < 0.01) and taurine, and correcting the energy metabolism disorder of tricarboxylic acid cycle. The study confirmed that the high-polarity metabolites of Gastrodia elata (T1) exerted the best therapeutic effect through anti-oxidation, anti-inflammation and repair of energy and amino acid metabolism disorders, in which water-soluble phenols and polysaccharides were the key active metabolites, providing a scientific basis at the metabolomic level for the application of Gastrodia elata in the treatment of gastritis (Xu et al., 2020).

## 5 The pharmacokinetics of Rhizoma Gastrodiae and its active metabolites

Detailed pharmacokinetic studies can well explain the pharmacokinetic characteristics of various active metabolites in GE *in vivo*. Understanding the pharmacokinetic characteristics of active metabolites in GE helps further investigate the interactions between GE and various chemical metabolites, and provides deeper insights into the influence of external factors on the efficacy of GE. Pharmacokinetic properties of *Gastrodia elata* metabolites are detailed in Table 7.

**TABLE 7:** The pharmacokinetics of Rhizoma Gastrodiae and its active metabolites.

Detected metabolites	Species	Route	Dose	Detection site	Cmax (μg/mL)	t1/2β (h)	AUC0− ∞ (μg/mL·h)	Method	Ref
Gastrodin	SD rat	i.g	1.7 g/kg	Plasma	Normal group:3.403 ± 0.623	Normal group:1.02 ± 0.42	Normal group:3.820 ± 0.830	UFLC–MS/MS	Guan et al. (2017)
					Migraine group:3.634 ± 0.369	Migraine group:1.31 ± 0.26	Migraine group:5.554 ± 1.754		
Gastrodin	Beagle dogs	i.g	0.125 g/kg	Plasma	1.20 ± 0.12	1.86 ± 0.67	5.080 ± 2.110	LC-ESI-MS/MS	Liu et al. (2017)
			0.25 g/kg		2.05 ± 0.495	1.90 ± 0.26	9.170 ± 1.790		
			0.5 g/kg		3.76 ± 0.778	2.09 ± 0.68	17.600 ± 4.710		
Parishin A			0.125 g/kg		0.388 ± 0.116	1.15 ± 0.18	0.899 ± 0.299		
			0.25 g/kg		0.462 ± 0.230	1.38 ± 0.34	1.520 ± 0.832		
			0.5 g/kg		0.969 ± 0.292	1.05 ± 0.16	3.280 ± 1.370		
Parishin B			0.125 g/kg		0.526 ± 0.143	1.08 ± 0.11	1.570 ± 0.533		
			0.25 g/kg		0.746 ± 0.320	1.18 ± 0.29	2.720 ± 1.140		
			0.5 g/kg		1.220 ± 0.562	1.17 ± 0.22	4.720 ± 2.330		
Parishin C			0.125 g/kg		0.107 ± 0.0294	1.37 ± 0.18	0.359 ± 0.099		
			0.25 g/kg		0.144 ± 0.0551	1.56 ± 0.41	0.578 ± 0.200		
			0.5 g/kg		0.243 ± 0.0996	1.29 ± 0.36	1.100 ± 0.527		
Parishin E			0.125 g/kg		0.136 ± 0.0353	3.09 ± 2.03	0.553 ± 0.190		
			0.25 g/kg		0.192 ± 0.0570	1.72 ± 0.13	0.868 ± 0.254		
			0.5 g/kg		0.312 ± 0.138	3.00 ± 1.71	1.630 ± 0.759		
NHBA	SD rat	i.g	0.2 g/kg	Plasma	0.108 ± 0.046	7.75 ± 2.83	0.434 ± 0.086	UPLC-QTOF-MS	Tang C. L et al. (2015)
Gastrodin	SD rat	i.g	0.3724 g/kg	Plasma	4.082	1.02	9.935	HPLC-MS/MS	Zhou et al. (2020)
Gastrodin	SD rat	i.g	40 mg/kg	Plasma	26.9 ± 5.3(Control group)	0.6 ± 0.0(Control group)	21.8 ± 2.3(Control group)	LC-MS/MS	Nepal et al. (2019)
					22.9 ± 5.5 (Antibiotic group)	0.6 ± 0.2 (Antibiotic group)	21.9 ± 1.4 (Antibiotic group)		
4-Hydroxybenzaldehyde	SD rat	i.g	40 mg/kg	Plasma	4.2 ± 1.0(Control group)	0.9 ± 0.3(Control group)	7.0 ± 1.4(Control group)		
					2.5 ± 0.8 (Antibiotic group)	1.1 ± 0.3 (Antibiotic group)	4.6 ± 1.0*(Antibiotic group)		
4-Hydroxybenzaldehyde	SD rat	i.g	400 mg/kg	Normal SD rat striatum	0.074 ± 0.017.00	11.29 ± 7.27	0.854 ± 0.293	LC-MS/MS	Feng et al. (2025)
				Normal SD rat cortex	0.011 ± 0.004	5.14 ± 1.67	0.071 ± 0.027		
				MCAO/R cortex	0.054 ± 0.005	22.73 ± 16.41	0.696 ± 0.348		
Adenosine	SD rat	i.g	4 mL	Plasma	—	5.37 ± 0.87	9.36 ± 1.04	HPLC	Bing et al. (2018)
4-Hydroxybenzyl alcohol						4.54 ± 0.69	16.3 ± 2.44		
Parishin C						5.34 ± 0.82	12.8 ± 1.57		
Gastrodin	SD rat	i.v	0.2 g/kg	Plasma	350.9 ± 56.1	0.687 ± 0.260	317.00 ± 113.35	HPLC-UV	Wang et al. (2008)
				Cerebrospinal flu	16.1 ± 7.7	1.078 ± 0.143	14.15 ± 6.68		
				Frontal cortex	21.6 ± 6.0	0.493 ± 0.317	9.80 ± 3.08		
				Hippocampus	24.3 ± 9.4	0.427 ± 0.188	9.16 ± 2.62		
				Thalamus	22.0 ± 6.9	0.463 ± 0.243	9.50 ± 3.14		
				Cerebellum	35.8 ± 10.3	0.420 ± 0.047	17.36 ± 4.32		
Gastrodin	SD rat	i.g	915 mg/kg	Plasma	55.73 ± 62.22	0.38 ± 0.16	115.22 ± 68.98	HPLC	Mi et al. (2020)
				Brain interstitial fluid	1.42 ± 0.24	0.53 ± 0.25	3.75 ± 0.63		
		i.g	GAS 915 mg/kg Ligustrazine 6.55 mg/kg Ferulic acid 79.7 mg/kg	Plasma	14.03 ± 6.92	0.48 ± 0.02	31.48 ± 13.7		
				Brain interstitial fluid	3.46 ± 0.13	0.60 ± 0.22	6.42 ± 0.25		
		i.g	GAS 915 mg/kg Ligustrazine 26.25 mg/kg Ferulic acid 315 mg/kg	Plasma	15.69 ± 1.75	0.69 ± 0.49 0.35 ± 0.16	27.23 ± 1.97		
				Brain interstitial fluid	6.96 ± 0.18		22.95 ± 1.53		

### 5.1 Absorption

Gastrodin was detectable in plasma within 10 min after intragastric administration to beagle dogs, with a time to peak concentration (Tmax) of 1.10–2.00 h, which prolonged with increasing doses. This is consistent with the rapid absorption observed in rats (detectable at 5 min post-gavage), though the Tmax was slightly longer (Tang C et al., 2015; Liu et al., 2017). This rapid absorption of gastrodin is primarily mediated by sodium-dependent glucose transporters (SGLTs). The SGLT inhibitor phlorizin (0.2 mM) significantly suppressed gastrodin absorption in rat perfused intestinal segments, reducing effective permeability to ∼30% in the duodenum and jejunum, and ∼10% in the ileum. In contrast, the facilitative glucose transporter (GLUT) inhibitor phloretin (0.2 mM) had no significant effect on gastrodin absorption, indicating that gastrodin uptake is predominantly mediated by SGLT1 rather than GLUT family transporters (Cai et al., 2013).

### 5.2 Distribution

Studies have shown that after intravenous injection of 100 mg/kg gastrodin in rats, it rapidly and widely distributes in a free state due to its water solubility, detectable in visceral tissues within 2 min. The brain-to-blood distribution ratio is only 0.007, leading researchers to suggest that the metabolic product of gastrodin, HBA, exerts therapeutic effects in the brain. HBA reaches peak cerebrospinal fluid concentration at 40 min post-oral administration, with a brain-to-blood ratio of ∼20%, indicating efficient blood-brain barrier (BBB) permeability. *In vitro* hCMEC/D3 model experiments show that 32.91% of HBA penetrates the barrier within 240 min, facilitated by its lipophilicity (XlogP3 = 0.2) and passive diffusion (Lin et al., 2007; Yan, 2024). This conclusion was later challenged: in a migraine rat model, oral gastrodin capsules increased AUC_0_‒ ∞ by 45.4%, decreased clearance (CL) by 28.3%, and prolonged Tmax by 74.1%, suggesting migraine may enhance gastrodin brain exposure by affecting BBB permeability or metabolic enzyme activity (Guan et al., 2017). Gastrodin exhibits higher AUC_0_‒ ∞ (1.042 ± 0.259 mg/mL·h) and Cmax (0.036 ± 0.010 mg/mL) in the cerebellum than in the frontal cortex, hippocampus, and thalamus, indicating preferential cerebellar targeting (Wang et al., 2008). Similarly, 4-hydroxybenzaldehyde shows selective brain distribution, with the highest concentration in the striatum of normal rats (Cmax = 0.074 μg/mL) and significantly increased cortical exposure (AUC = 0.696 mg/mL·h) with prolonged retention (t_1_/_2_β = 22.73 h) in MCAO/R models, suggesting ischemia enhances its brain targeting (Feng et al., 2025). Traditional Gastrodia elata decoctions often combine with other botanical drugs (e.g., Ligusticum chuanxiong), whose active metabolites ferulic acid and ligustrazine improve gastrodin brain distribution. In low-dose ligustrazine and ferulic acid groups, interstitial fluid Cmax of GAS increased by 2.36-fold (314.33 ± 14 vs. 132.95 ± 4.08 μg/mL), and high-dose combination further elevated AUC by 6.12-fold (1377.26 ± 92.16 vs. 224.98 ± 37.54 μg/mL·min), indicating synergistic effects of traditional formulations (Mi et al., 2020). Other active metabolites show characteristic tissue distributions: adenosine peaks in the spleen (0.678 mg/mL at 4 h), followed by the lung, with negligible kidney and brain levels; 4-hydroxybenzyl alcohol accumulates in the liver; Parishin C peaks in the heart (4 h), followed by the liver and spleen, with low brain and lung concentrations. These distributions partially explain adenosine’s immunomodulatory role, 4-hydroxybenzyl alcohol’s hepatic detoxification, and Parishin C’s cardioprotection as reported in literature (Guan et al., 2017).

### 5.3 Metabolism

In phase I metabolism mediated by cytochrome P450 (CYP) enzymes, rats and dogs showed higher metabolic capacity for gastrodin, with remaining amounts of 29% and 24%, respectively, while humans and monkeys exhibited weaker metabolism (remaining amounts. 70% and 71%). In phase II metabolism mediated by uridine diphosphate glucuronosyltransferase (UGT), the remaining amounts in dogs and rats were 34% and 42%, compared to 67% and 63% in humans and monkeys. Molecular docking showed that gastrodin binds to CYP3A4 and CYP2C19 via hydrogen bonds, confirming these enzymes as potential major metabolic enzymes (*in vitro* metabolic differences of gastrodin in liver microsomes of different species detected by high-performance liquid chromatography). Stability of results also influences metabolism: compared to gastrodin, N^6^-hydroxybenzyladenosine has a significantly longer half-life (gastrodin t_1_/_2_β: ∼1.86–2.09 h), likely attributed to the metabolic stability of its nucleoside structure (determined by ultra-high-performance liquid chromatography-quadrupole time-of-flight mass spectrometry for N^6^-hydroxybenzyladenosine in rat plasma). Intestinal microbiota affect metabolism: after oral administration, gastrodin is rapidly absorbed, with no significant differences in Cmax and AUC between control and antibiotic groups. However, pharmacokinetic parameters of the gastrodin metabolite 4-HBA changed significantly: antibiotic treatment reduced 4-HBA Cmax by 40.5% and AUC by 34.3%, while volume of distribution (Vd) and clearance (CL) increased by 100% and 55.2%, respectively, indicating that intestinal microbiota inhibition significantly reduces conversion of gastrodin to 4-HBA (Nepal et al., 2019). Pharmacokinetic parameters (e.g., Tmax, AUC) of Parishin metabolites (e.g., Parishin B, C, E) differ from those of gastrodin. Pearson correlation coefficients between Parishin A and Parishin B/C/E range from 0.76 to 0.95 (e.g., 0.95 for Parishin A and E), significantly higher than those with gastrodin (0.54–0.57), suggesting that Parishin metabolites may interconvert via metabolism, whereas gastrodin shows weak correlation with Parishins, possibly exerting effects through different metabolic pathways (Liu et al., 2017).

### 5.4 Excretion

Gastrodin is primarily excreted in urine as the parent metabolite. In normal rats, the clearance (CL) of gastrodin after oral administration of 200 mg/kg was 5.73 ± 1.59 mL/min, indicating rapid elimination (Cai et al., 2013; Lin et al., 2007). In diabetic rats with upregulated intestinal sodium-dependent glucose transporter 1 (SGLT1) expression, the Tmax of gastrodin was significantly shortened to 20.0 ± 0.0 min, suggesting accelerated absorption. However, the elimination rate constant (k) was 0.033 ± 0.013 L/min, not significantly different from normal rats (0.040 ± 0.014 L/min), indicating that increased SGLT1 expression did not alter elimination rate (Cai et al., 2013). When comparing single extract and metabolite formulations, the elimination rate constant (Ke) of gastrodin in rats administered pure Gastrodia elata extract was 0.0204 ± 0.004 min^-1^. In the Tian Gou Jiang Ya Capsule metabolite formulation, Ke decreased to 0.017 ± 0.001 min^-1^, 0.0151 ± 0.003 min^-1^, and 0.013 ± 0.001 min^-1^ with increasing doses (low, medium, high), indicating that the metabolite formulation delayed gastrodin elimination and prolonged its residence time *in vivo*. Although the metabolite formulation altered elimination rate and distribution of gastrodin, the area under the curve (AUC) showed no significant difference from the single extract: the medium-dose metabolite group had an AUC of 5187.2 ± 871.5 μg/mL, compared to 5462.1 ± 281.2 μg/mL in the extract group, without statistical significance. This suggests that the metabolite formulation did not significantly affect the total absorption of gastrodin, only altering its elimination kinetics (Cai et al., 2013; Song et al., 2017).

## 6 Clinical efficacy of marketed preparations of Gastrodia elata

Gastrodia elata has demonstrated definite therapeutic effects, leading to the successive market launch of related products. The only marketed monomeric drugs are gastrodin and its derivative acetylgastrodin, while other products are formulations prepared from Gastrodia elata extracts combined with other botanical drugal medicines. As of 3 July 2025, a search of China’s medical information platform using “Gastrodia elata” as a keyword identified 163 registered preparations and related formulations (China Medical Information Platform, 2025). Clinical studies on these preparations primarily focus on cardiovascular and cerebrovascular diseases and neuroprotection. Most studies adopt a placebo-controlled design, while a few use positive drug controls to observe enhanced efficacy. The corresponding clinical effects are shown in Table 8. The following clinical comparative experiments confirm the efficacy of Gastrodia elata preparations.

**TABLE 8:** Clinical efficacy of marketed preparations of Gastrodia elata.

Study subjects	Treated diseases	Dosage administered	Control drug	Clinical phase	Treatment duration	Number of cases(n)	Evaluation indicators	Treatment outcomes	Ref
Total extract of Gastrodia elata	Vascular dementia (VaD)	TMBCZG 3 tab, bid TMBCZG 1 tab + Placebo 2 tab, bid TMBCZG 2 tab + Placebo 1 tab, bid Placebo 3 tab, bid	Placebo	IIa	24 weeks	40/40/40/40	Vascular Dementia Assessment Scale-Cognitive Clinical Dementia Rating-Sum of Boxes	Results not yet published	Tian et al. (2018)
Tianma gouteng granules	Masked hypertension	Initial dose: 5 g bid After 2 weeks, titrate to 10 g bid	Placebo	IIa	8 weeks	126/125	Magnitude of blood pressure decrease	Daytime blood pressure reduction: GUG group vs. placebo group Systolic blood pressure: 5.44 vs. 2.91 mmHg (between-group difference 2.52 mmHg, P = 0.025) Diastolic blood pressure: 3.39 vs. 1.60 mmHg (between-group difference 1.79 mmHg, P = 0.011) Proportion of daytime systolic reduction ≥10 mmHg or diastolic reduction ≥5 mmHg 44.4% in GUG group vs. 29.6% in placebo group	Zhang et al. (2020)
Qizhitongluo capsule	Lower limb motor dysfunction after ischemic stroke	QZTL group: 4 cap/dose (500 mg/cap) (after breakfast and dinner), Placebo: 4 cap (after lunch), total daily dose 4000 mg 1.6 g/d, po tid + riluzole (100 mg/d) 2.4 g/d, po tid + riluzole (100 mg/d)	NXT group: 4 cap/dose (400 mg/cap), tid (After meals), total daily dose 4000 mg. Placebo: 4 cap/dose, tid (After meals)	Not mentioned (Multicenter Trial)	12 weeks	QZTL: 309 NXT: 159 Placebo: 154	Change in Lower Limb Fugl-Meyer Motor Scale (FMMS-LL) score from baseline to 12 weeks	QZTL group: +4.81 points from baseline at 12 weeks (95% CI 4.27–5.35) NXT group: +3.77 points (95% CI 3.03–4.51) Placebo group: +3.00 points (95% CI 2.24–3.76) Placebo group: −2.78 points from baseline at 12 weeks Mecasin 1.6 g group: −0.25 points from baseline, 2.53 points less decrease than placebo group (95% CI: 0.61–4.45, P < 0.05) Mecasin 2.4 g group: −1.32 points from baseline, 1.46 points less decrease than placebo group (95% CI: 0.48–3.40, P < 0.05)	Yu et al. (2021)
Mecasin (Compound preparation of Gastrodia elata)	Amyotrophic Lateral Sclerosis, ALS		Placebo + riluzole (100 mg/d)	IIa	12 weeks	10/10/10	Change in Korean Amyotrophic Lateral Sclerosis Functional Rating Scale-Revised (K-ALSFRS-R) score		Kim et al. (2023)
Gastrodin	Postoperative Delirium and Postoperative Cognitive Dysfunction (POCD) after cardiac surgery	600 mg/50 mL 0.9% Nacl, bid, iv 1 h, total daily dose 1200 mg	Placebo	Not mentioned	7 days	77/78	Incidence of postoperative delirium	Incidence of delirium in gastrodin group was significantly lower than placebo group (19.5% vs. 35.9%), relative risk (RR) 0.54 (95% CI 0.32–0.93, p = 0.022)	Bai et al. (2025)
							Incidence of postoperative cognitive dysfunction	Incidence of POCD showed no significant difference between groups	
Gastrodin	Cognitive decline after cardiopulmonary bypass cardiac surgery	40 mg/kg dissolved 50 mL 0.9% Nacl, iv after anesthesia induction, 45 min	Placebo	Not mentioned	Once	100/100	Incidence of cognitive decline before discharge	Before discharge: incidence of cognitive decline was 9% in gastrodin group, significantly lower than 42% in control group (P < 0.01)	Zhang et al. (2011)
							Incidence of cognitive decline 3 months after surgery	At 3 months postoperation: incidence of cognitive decline was 6% in gastrodin group, significantly lower than 31% in control group (P < 0.01)	
Gastrodin	Refractory hypertension in the elderly	Routine antihypertensive drugs +1000 mg gastrodin, iv gtt, qd	Routine antihypertensive drugs (one or more of amlodipine, irbesartan, hydrochlorothiazide)	Not mentioned	4 weeks	33/30	Blood pressure changes	No change in diastolic blood pressure	Zhang et al. (2008)
							ET/NO (Endothelin/Nitric Oxide)	Systolic blood pressure decreased steadily with prolonged treatment: average 12 mmHg reduction after 2 weeks (*P* = 0.005); ET decreased, NO increased	

## 7 Conclusion

Despite the fact that *Gastrodia elata* has been researched and used for thousands of years in China, its level of utilization remains relatively low. Among the five commonly used medicinal varieties of Rhizoma Gastrodiae, only two types are widely cultivated and utilized (Zhan et al., 2016; Zhengyi, 1999). At present, the primary method of using *Gastrodia elata* is still traditional decoction, and related formulations mainly consist of traditional Chinese medicine, with only gastrodin being used in clinical single-agent preparations (Tian et al., 2022; Kong et al., 2022). This situation is not conducive to the utilization and modernization of Rhizoma Gastrodiae, indicating a need for further research to fill the gap. The reason for this situation is the insufficient research on the extracted metabolites. Transcriptomics (RNA-seq) and targeted metabolomics (HPIC-MS/MS) technologies can be used to systematically explore the therapeutic mechanisms of other active metabolites of Gastrodia elata in diseases, which is conducive to the development of monomer drug research (Song et al., 2024).

Through literature surveys, it has been found that more than a hundred metabolites have been extracted from Rhizoma Gastrodiae to date, with the most extensively studied being gastrodin, which is the main active substance known to treat hypertension, headaches, and other brain-related injuries. It has been shown to exhibit significant antioxidant, anti-aging, and neuroprotective effects (Kong et al., 2022; Yu, 2022). However, there has been relatively little research on other metabolites such as polysaccharides, sterols, and organic acids. Although some studies have confirmed that such metabolites possess certain pharmacological effects (Zhan et al., 2016; Lai et al., 2017; Abdel-Salam et al., 2014; Chen et al., 2016; Chen et al., 2015), the evidence in this area still suggests that the pharmacological research on *Gastrodia elata* remains limited. Through multi-omics integration, such as transcriptomics (RNA-seq) and targeted metabolomics (HPIC-MS/MS), combined with KEGG and GO enrichment analyses, differentially expressed genes following administration of such metabolites can be identified to investigate how other metabolites in Gastrodia elata exert therapeutic effects by regulating gene expression (Song et al., 2024; Kim et al., 2022).

Regarding the research on *Gastrodia elata*, the specific parts of the plant also need to be considered. Ancient medical texts in China mainly focus on the utilization of the tuber, and current studies primarily investigate the extracts from the tuber. Research on the leaves, flowers, and stems of GE is relatively scarce (Martins and S, 2018; Hu et al., 2019). Our comparative analysis of three Gastrodia elata variants (GE Bl. f. elata, GE Bl. f. viridis, and GE Bl. f. glauca S. Chow) revealed significant differences in metabolite composition between stems and tubers (Table 1). The stems exhibited greater chemical diversity, containing 128 identified metabolites compared to 90 in tubers, with 80 metabolites shared between both parts (Wu et al., 2023). The quantitative analysis reveals significant variation in gastrodin content across different plant parts of Gastrodia elata Blume f. elata. Specifically, the fresh stem bark exhibits the highest concentration at 0.640%, followed by fruits (0.302%) and seeds (0.094%). Notably, both fresh stem barks and fruits meet the quality standard stipulated in the Chinese Pharmacopoeia (2020 edition), which mandates a minimum combined content of 0.25% for gastrodin and p-hydroxybenzyl alcohol. This compliance suggests these morphological parts possess adequate pharmacological potency for medicinal applications. The three-fold difference between stem bark (0.640%) and seed (0.094%) concentrations may reflect distinct biosynthetic activity or metabolite translocation patterns during plant development, warranting further phytochemical investigation (Commission ChP, 2020; Li, 2012). The comparative analysis of both phytochemical composition and pharmacological content reveals that non-tuber parts of GE (including stems, leaves, and flowers) possess considerable medicinal potential. Historically, these aerial portions have been underutilized in traditional Chinese medicine, as evidenced by the current Chinese Pharmacopoeia standards which exclusively regulate quality parameters for the tuberous rhizomes (Commission ChP, 2020). This phenomenon could be attributed to multiple historical and technological factors: (1) In ancient times, the absence of proper processing techniques for stem barks, (2) limited pharmacological understanding of stem bark metabolites, and (3) the empirically verified superior therapeutic efficacy of tubers compared to other plant parts through millennia of clinical practice. Modern analytical advancements—particularly the integration of ultra-high performance liquid chromatography-tandem mass spectrometry (UPLC-MS/MS) with high-performance liquid chromatography-ultraviolet detection (HPLC-UV) metabolomic platforms—enable comprehensive phytochemical profiling. These technologies not only elucidate compositional differences in bioactive metabolites among Gastrodia elata’s anatomical parts, but also establish metabolomic foundations for: (a) cultivar authentication, (b) quality standardization, and (c) pharmaceutical development of this medicinal species (Zeng et al., 2023).

In general, studies on gastrodia metabolites have found many different effects from traditional uses of *Gastrodia elata*, including improving immunity, anti-tumor, and delaying aging (Farooq et al., 2019; Liang et al., 2017). However, the research is relatively limited, and only gastrodin is widely accepted and used clinically (Kong et al., 2022). On the other hand, a critical gap is the overreliance on preclinical data: while antitumor and anti-osteoporosis effects show promise in in vitro (e.g., gastrodin in DBTRG-05MG cells) and *in vivo* models (e.g., WSS25 in ovariectomized mice), the absence of human clinical trials—particularly for postmenopausal osteoporosis—severely undermines their clinical relevance. Additionally, mechanistic insights into some active components are incomplete. Although β-sitosterol’s anti-inflammatory role via NF-κB pathway modulation is established, its precise binding sites (e.g., on p65) and molecular interactions remain undefined. Likewise, research on gastrodin polysaccharides and gut microbiota lacks definitive evidence connecting microbial structural shifts to immune improvement, resulting in unresolved mechanistic pathways. Therefore, other active metabolites of gastrodia have not been fully researched yet, leaving plenty of room for further exploration. In-depth research on other gastrodia metabolites can further explore their potential biological activities, functions, and applications.

## Glossary

5-HT: 5-Hydroxytryptamine
6-OHDA: 6-Hydroxydopamine hydrobromide
A/R: Anoxia/Reoxygenation
ACSL4: Acyl-CoA synthetase long-chain family member 4
AF4: Asymmetrical Flow Field-Flow Fractionation
APP: Amyloid β precursor protein
BDNF: Brain derived neurotrophic factor
BMP-2: Morphogenetic protein 2
CDK1/CDC2: Cyclin-dependent kinases 1/2
c-eEPSCs: C-fiber evoked EPSCs
CGRP: Calcitonin gene-related peptide
CIRI: Cerebral ischemia-reperfusion injury
COX2: Cyclooxygenase-2
DA: Dopamine
dRI: Differential refractive index
EPSCs: Excitatory postsynaptic currents
FPN1: Ferroportin 1
GABA: γ-aminobutyric acid
GE: Gastrodia elata
GSDMD: Gasdermin D
HBA: p-Hydroxybenzyl alcohol
HDL-C: High cholesterol
HPA: Hypothalamic-pituitary-adrenal
I/R: Ischemia-reperfusion
ICAM-1: Intercellular cell adhesion molecule-1
IDO 1: Indoleamine 1
ISVs: Intersegmental vessels
KA: Kainic acid
LDL-C: Lipoprotein cholesterol
LPS: Lipopolysaccharide
LTP: Long-term potentiation
MALS: Multi-angle light scattering
MAO-A: Monoamine oxidase
MCAO: Middle cerebral artery occlusion
MCP-1: Macrophage chemotactic protein-1
MDA: Malondialdehyde
MPO: Myeloperoxidase
MT1/2: Melatonin 1/Melatonin 2
NFATc1: Nuclear factor of activated T cells cytoplasmic 1
NFTs: Neurofibrillary tangles
NHBA: N^6^-(4-hydroxybenzyl) adenine riboside
NLRP3: NOD-like receptor thermal protein domain associated protein 3
NMDAR: N-methy-D-aspartate receptors
NSCs: Neural stem cells
NTG: Nitroglycerin
OCN: Osteocalcin
OGD/R: Oxygen-glucose deprivation reperfusion
ROS: Reactive oxygen species
SHR: Spontaneously hypertensive rats
TBI: Traumatic brain injury
TC: High cholesterol
TH: Tyrosine hydroxylase
TPH 2: Tryptophan hydroxylase 2
UCMS: Unpredictable chronic mild stress
Vanillin: 4-hydroxy-3-methoxybenzaldehyde
VCAM-1: Vascular Cell Adhesion Molecule 1
WGEW: Water-soluble polysaccharide extracted from Gastrodia elata
WSS25: A sulfated polysaccharide extracted from the rhizome of Gastrodia elata that binds to bone morphogenetic protein 2
